# Potential Role of MAP3K14 in Hepatocellular Carcinoma: A Study Based on Comprehensive Bioinformatical Analysis and Validation

**DOI:** 10.7150/jca.95322

**Published:** 2024-03-17

**Authors:** Ke Tang, Weiquan Lin, Dedong Wang, Xiangzhi Hu, Zhitao Chen, Jinbin Chen, Boheng Liang, Lin Zhang, Pengzhe Qin, Di Wu

**Affiliations:** 1 School of Public Health, Guangdong Medical University, Dongguan City, 523808, China.; 2 Guangzhou Center for Disease Control and Prevention, Guangzhou City, 510440, China.; 3 Department of Public Health and Preventive Medicine, School of Medicine, Jinan University, Guangzhou City, 510632, China.; 4 Guangzhou key laboratory for clinical rapid diagnosis and early warning of infectious diseases, KingMed School of Laboratory Medicine, Guangzhou Medical University, Guangzhou City, 510180, China.; 5 The State Key Lab of Respiratory Disease, School of Public Health, Guangzhou Medical University, Xinzao Town, Panyu District, Guangzhou, 511436, China.

**Keywords:** hepatocellular carcinoma, MAP3K14, prognosis

## Abstract

According to reports, MAP3K14 is considered an oncogene and is aberrantly expressed in various types of tumor cells. Its abnormal expression is closely associated with the occurrence and progression of various cancers. MAP3K14 also plays a significant role in the development of non-alcoholic steatohepatitis (NASH)-related hepatocellular carcinoma and its connection to tumor stem cells. The prognostic value of MAP3K14 in HCC, as well as its potential functions and roles, requires further elucidation. We evaluated the potential role of MAP3K14 in HCC based on data mining from a range of public databases. The bioinformatics analysis of TCGA, GEO, TIMER, cBioportal, Kaplan-Meier plotter, MethSurv, ENCORI and CellMiner databases was carried out. The expression of MAP3K14 protein in HCC was detected by immunohistochemical method. The mRNA and protein expression levels of MAP3K14 in tumor tissues were higher than those in normal tissues (p < 0.05). The expression of MAP3K14 was correlated with Pathologic T stage (p=0.026), Pathologic stage (p=0.032), Tumor status (p=0.024) and AFP (p=0.002). HCC patients with high expression of MAP3K14 had poor overall survival (OS), progression free survival (PFS) and recurrence free survival (RFS). Multivariate Cox regression analysis showed that the Pathologic stage (p < 0.001) and MAP3K14 expression levels (p < 0.05) is an independent prognostic factor affecting the survival of patients with liver cancer. GO/KEGG analysis suggested that key biological processes (PI3K-Akt signaling pathway) may be the mechanism promoting HCC development. In addition, MAP3K14 was significantly correlated with the infiltrating levels of B cells, CD8+ T cells, CD4+ T cells, macrophages, neutrophils, and dendritic cells (p < 0.05). MAP3K14 is up-regulated in HCC and is closely related to the prognosis of HCC patients. MAP3K14 may serve as a potential biomarker for poor prognosis of HCC.

## 1. Introduction

According to the Global Cancer 2020 data, liver cancer has emerged as the sixth most prevalent type of cancer and the third leading cause of cancer-related deaths worldwide. Notably, China accounts for almost half of all liver cancer cases reported globally 1. Hepatocellular carcinoma (HCC) is the most common histological type of liver cancer. It is a clinical challenge and aggressive malignancy, being one of the most common and deadly malignant tumors affecting the digestive system 2. It has caused significant economic and health burdens worldwide. Chronic liver disease resulting from hepatitis B virus (HBV) and hepatitis C virus (HCV) infection is the leading cause of hepatocellular carcinoma (HCC). Excessive alcohol consumption and non-alcoholic fatty liver disease, which is linked to metabolic syndrome, are also significant contributors to HCC 3, 4. Radical therapeutic measures, such as liver transplantation, surgery, and ablation, have proven to be effective treatments for early-stage hepatocellular carcinoma 5. Most patients typically undergo surgical resection or liver transplantation as the primary treatment options. However, it is important to note that even after surgery, there is a risk of recurrence, metastasis, or graft rejection. Unfortunately, the 5-year survival rate for patients who experienced recurrence is approximately 30%, highlighting the limited effectiveness of current treatments 6. In addition to the challenge of early diagnosis of HCC, many HCC patients are typically diagnosed at advanced stages and exhibit highly aggressive behavior. This significant factor contributes to the high mortality rate observed among patients 3. In recent years, significant advancements have been achieved in the treatment of HCC, encompassing surgical procedures, chemotherapy, and targeted therapy. Despite these advancements, the 5-year survival rate for HCC remains alarmingly low 7. In recent years, multi-omics studies have demonstrated the intricate and multifaceted nature of HCC development and progression, involving numerous genetic and epigenetic alterations. The discovery of novel indicators or biomarkers for HCC holds potential for improving patient treatment and enhancing our understanding of the underlying mechanisms. Hence, the quest for new HCC markers is crucial for accurate diagnosis and prognosis of HCC.

Mitogen-activated protein kinase kinase 14 (*MAP3K14*), also known as NF-κB-inducible kinase or NIK, is located on the 17th chromosome in humans and is crucial in the nonclassical pathway 8, 9. Regulation of NIK activity primarily occurs at the post-translational level. Overexpression of NIK is associated with metabolic disorders, inflammatory diseases, as well as cancer development and progression 10. Aberrant expression of NIK has been reported in a variety of cancer types, including hematologic and solid cancers 10, 11. As reported by Hayashi Y et al., abnormal accumulation of NIK was found to promote tumor growth in a highly malignant breast cancer cell line 12.

However, there are limited studies reporting the involvement of *MAP3K14* in HCC and its prognostic value. Therefore, this study aims to analyze the expression level of *MAP3K14* and its prognostic significance in hepatocellular carcinoma using data mining from multiple databases. The findings of this study can potentially serve as a valuable basis for clinical diagnosis and treatment.

## 2. Materials and Methods

### 2.1 TCGA data download, process, and analysis

Transcriptome sequencing data and detailed clinical data of patients with hepatocellular carcinoma (TCGA-LIHC) were searched and downloaded from the Cancer Genome Atlas (TCGA) database 13. After removing duplicate information, a total of 374 HCC cases and 50 normal cases were included. The gene expression data was standardized and transformed into TPM (transcripts per million) for subsequent analysis. This transformation to TPM is advantageous as it produces results that are more comparable between samples, similar to the results obtained from microarray methods. In addition, two microarray gene expression datasets were obtained from the Gene Expression Omnibus (GEO) database 14, including GSE144269 and GSE64041.These data were subsequently normalized and then subjected to differential expression analysis using R-4.2.2, and *p* < 0.05 were considered statistically significant.

### 2.2 UALCAN database analysis

UALCAN is an online public portal that provides in-depth analysis of TCGA data. In the present study, this database was used for gene expression and clinical characteristics subgroup analysis 15, and *p* < 0.05 were considered statistically significant.

### 2.3 Kaplan-Meier plotter database analysis

The Kaplan-Meier plotter is an online database with access to the impact of genes or miRNAs on survival in more than 20 cancer types, including hepatocellular carcinoma 16. It was used for survival analysis of *MAP3K14* in hepatocellular carcinoma.

### 2.4 Immunohistochemical (IHC) analysis of MAP3K14 protein in HCC tissue

To validate the results from TCGA and GEO databases, we performed IHC analysis using the HCC tissue microarray (product code: HLivH180Su11) provided by Shanghai Outdo Biotech Co., Ltd. the patients involved in study were recruited by the Shanghai Outdo Biotech Co., Ltd., and the analysis was also conducted and approved by the ethics committee of Shanghai Outdo Biotech Co., Ltd (ethical protocol code SHYJS-CP-1701002 on January 6, 2017). A total of 94 cases histologically diagnosed with HCC were included in this study, consisting of 94 cancerous tissue samples and 86 adjacent non-cancerous tissue samples. These patients underwent surgery between January 2007 and November 2009 and were followed up for 4-6.7 years.

The immunohistochemistry experimental protocol is as follows:Place the tissue chips in a 63-degree oven for one hour to wax them. After dewaxing in an automated staining machine (model: LEICAST5020), perform antigen retrieval in a citrate buffer solution. Incubate the sections with anti-NFKB antibodies (ab228587; diluted 1:500) at 4 ℃ overnight. Followed by secondary antibody incubation (kit: EnVision™ FLEX+, Mouse, High pH, (Link), catalog number: K8002). Retrieve the slides from the refrigerator, allow them to equilibrate at room temperature for 45 minutes, and rinse with phosphate-buffered saline (PBS). Finally, perform 3,3-diaminobenzidine tetrahydrochloride (DAB) staining, counterstain with hematoxylin, dehydrate, and mount with neutral resin.The following categories have been defined for evaluation: (i) Cell staining intensity is graded on a scale of 4, with no positive staining (negative) scored as 0, pale yellow (weak positive) scored as 1, brownish-yellow (positive) scored as 2, and brown (strong positive) scored as 3; (ii) Percentage of positive cells is graded on a scale of 4, with ≤25% scored as 1, 26%-50% scored as 2, 51%-75% scored as 3, and >75% scored as 4. The final score is obtained by multiplying the scores from these two evaluations (score range: 0-12). Scores within the range of 0-6 are considered indicative of negative or low expression of MAP3K14, while scores within the range of 7-12 indicate high expression of MAP3K14.

Based on the results of the immunohistochemistry experiment, we excluded samples that did not yield any experimental results due to reasons such as tissue detachment. This included 8 hepatocellular carcinoma tissue samples and 7 normal tissue samples. Ultimately, we included 86 hepatocellular carcinoma tissue samples and 79 normal tissue samples for differential expression analysis. According to the aforementioned scoring criteria, the 86 hepatocellular carcinoma tissue samples were divided into a high-expression group and a low-expression group for further analysis.

### 2.5 Protein‐protein interactions of *MAP3K14*

GeneMANIA can be used to search large publicly available biological datasets for genetic interactions of proteins, protein-DNA, and related genes 17. We selected the default species Homo sapiens and utilized GeneMANIA to construct an online protein-protein interaction (PPI) network. This network analysis was conducted to explore the interactions between *MAP3K14* and other functional proteins.

### 2.6 Human Protein Atlas (HPA) analysis

The Human Protein Atls (HPA) is a public database containing large-scale human protein profiles 18, The Human Protein Atlas is utilized for gene retrieval in different cell types, as well as for accessing clustered homogeneous flow approximation and projection maps and expression histograms. In our study, we investigated the expression patterns of genes such as *MAP3K14*, which serve as clustered cell markers, across various single-cell types.

### 2.7 Screening of MAP3K14 co‐expressed genes in HCC and functional analysis

The Gene expression profiling interactive analysis (GEPIA2) database provides customizable features for analyzing tumor and normal differential expression, as well as detecting similar genes and performing correlation analysis 19. To investigate the potential biological functions of *MAP3K14* in the pathogenesis and progression of HCC, we utilized the GEPIA2 database to search for the top 200 genes highly associated with *MAP3K14*. Subsequently, these genes were subjected to GO and KEGG enrichment analysis using the "clusterProfiler" package in R language, and functional annotation tables were generated.

### 2.8 TIMER database analysis

The Tumor Immune Estimation Resource (TIMER) database 20 is used to explore the relationship between gene expression, mutations, and immune cell infiltration levels. The "gene" module analysis in TIMER reveals the association between *MAP3K14* expression levels and immune cell infiltration levels. This analysis can be utilized to investigate the correlation between *MAP3K14* expression levels and the levels of immune cell infiltration as well as immune checkpoint expression in HCC. *p* < 0.05 was considered statistically significant.

### 2.9 Alternation of MAP3K14 in HCC

Malignant tumorigenesis and progression are influenced by mutations in specific regulatory genes, cBioportal database 21 can visualize patterns of genetic alterations among multiple samples in a cancer study and compare the frequency of genetic alterations in point-multiple cancer studies, or summarize an overview of all relevant genomic alterations in an individual tumor sample. Based on data filtering and extraction conditions, we investigated the type and frequency of *MAP3K14* mutations in HCC.

### 2.10 Correlation between methylation and MAP3K14 expression

DNA methylation of gene promoters plays a crucial role in regulating gene expression and influencing the development of diseases. We utilized the DNA Methylation Interactive Visualization Database (DNMIVD) 22 and the MethSurv database 23 to retrieve methylation sites within the *MAP3K14* promoter region. Additionally, we obtained methylation data from HCC patients by downloading the relevant data from the UCSC Xena database 13. Through further analysis of these datasets, we compared the differences in methylation status at the *MAP3K14* promoter region between normal samples and HCC patient samples.

### 2.11 Construction of the lncRNA-miRNA-mRNA triple regulatory network

We used the miRWalk 24 and ENCORI 25 databases to predict miRNAs targeting *MAP3K14*. We filtered out miRNAs with differential expression and favorable prognosis. Finally, we determined the subcellular localization of the lncRNAs using the lncLocator database 26.

### 2.12 Drug susceptibility analysis

The CellMiner 27 database helps researchers integrate and analyze molecular and pharmacological data from the NCI-60 cancer cell line.The database allows rapid searching of transcripts for 22,379 genes and 360 microRNAs, as well as activity reports for 20,503 compounds, including 102 drugs approved by the U.S. Food and Drug Administration. Clinically tested and FDA-approved drugs were selected for drug susceptibility analysis in this study. The data processing and plotting were performed using the "impute", "limma", and "ggpubr" packages in R.

### 2.13 Statistical analysis

The statistical analysis was performed using R software (v.4.2.2) and GraphPad Prism (version 8.0). The results are presented as median values with 95% confidence intervals (CI). Kaplan-Meier method was employed for survival analysis, and group comparisons were conducted using the log-rank test. Furthermore, the relationship between clinicopathological features and survival was investigated using the Cox regression model. Differences between groups were evaluated using Mann-Whitney U test, independent samples t-test, and chi-square test. In all analyses,* p* < 0.05 was considered statistically significant. *, **, and *** indicated* p* < 0.05, *p* < 0.01, and *p* < 0.001, respectively.

## 3. Results

### 3.1 Pan-cancer analysis of MAP3K14 expression

**Figure 1:**
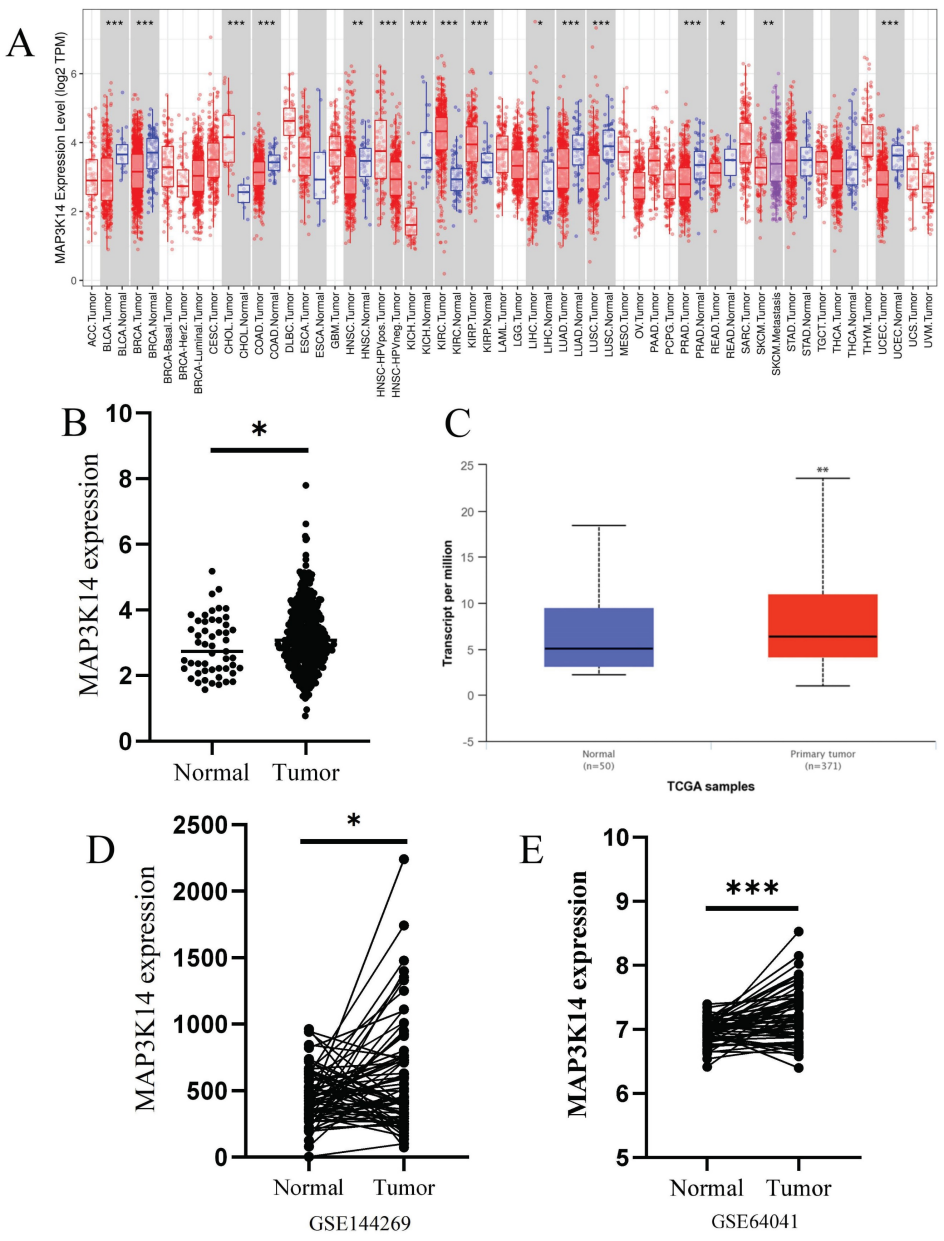
Relationship between *MAP3K14* expression and hepatocellular carcinoma (HCC). (A) Transcription expression of *MAP3K14* in 37 distinct cancer types (TCGA). (B-C) The expression of *MAP3K14* in HCC tissues and normal tissues based on TCGA. (D-E) *MAP3K14* expression in HCC tissues and normal tissues based on GSE64041, GSE144269. *MAP3K14*: Mitogen-Activated Protein Kinase Kinase Kinase 14; TCGA: The Cancer Genome Atlas.

Firstly, the expression levels of *MAP3K14* in different tumor tissues and adjacent tissues were examined using the Timer database. As shown in Figure 1A, *MAP3K14* is highly expressed in various malignant tumors, including HCC. To explore the expression of *MAP3K14* in HCC, the Transcriptome data of HCC were retrieved from TCGA database. The expression level of *MAP3K14* in HCC tissue was significantly higher than that in normal tissue, and the above results were validated using the UALCAN database (*p* < 0.05, *p* < 0.01, Figure 1B, C). Additionally, analysis of the GSE144269 and GSE64041 datasets also confirmed that *MAP3K14* was upregulated in HCC tissues compared to normal liver tissues (*p* < 0.05, *p* < 0.01, respectively, Figure 1D, E).

### 3.2 Relationship of mRNA Levels of MAP3K14 and clinicopathological features of HCC patients

**Figure 2:**
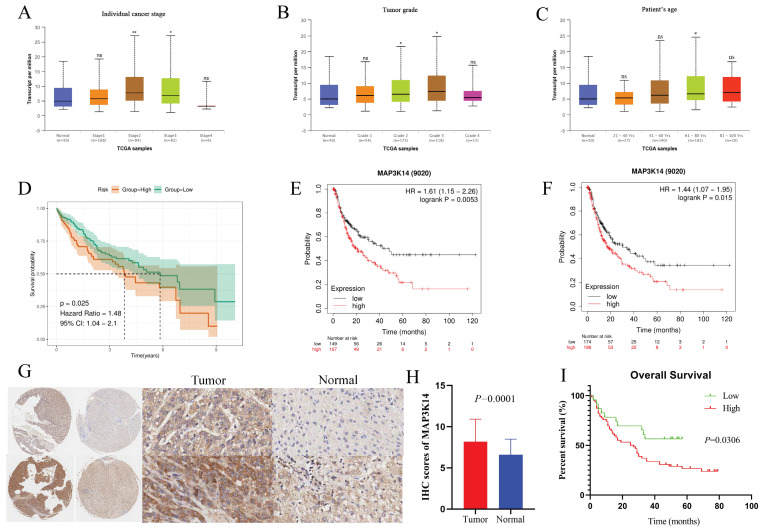
Correlation between *MAP3K14* expression and the clinical parameters of HCC patients and its prognostic significance. (A-C) Relationship of *MAP3K14* mRNA levels with individual cancer stages, tumor grade, and age of HCC patients. (D) Relationship between *MAP3K14* expression and OS in the TCGA database. (E-F) Association between *MAP3K14* expression and PFS and RFS in HCC patients in the Kaplan-Meier Plotter database. (G) The expression of *MAP3K14* protein was examined in two pairs of HCC tissues and adjacent non-cancerous tissues. (H) The histogram depicts the protein level expression scores of *MAP3K14* in HCC and non-cancerous tissues. (I) The association between *MAP3K14* expression and overall survival (OS) in HCC patients. HCC: hepatocellular carcinoma; RFS: Recurrence Free Survival.

UALCAN was used to assess the relationship between *MAP3K14* expression and the clinicopathologic features of HCC patients, including cancer stage, tumor grade and patient age. The highest mRNA levels of *MAP3K14* were predominantly found in patients in stages II and III (Figure 2A). Tumor pathologic grade has important prognostic significance and patients with high-grade tumors tended to exhibit higher *MAP3K14* mRNA levels (Figure 2B). *MAP3K14* expression was significantly higher, mainly in the age group of 61-80 years (*p* < 0.05), with higher mRNA expression levels likely at higher ages (Figure 2C). To verify the prognostic value of *MAP3K14* expression in HCC cases, HCC cases were collected from the TCGA database. Using the A function of the R language "surveyor" package divides patients into high expression and low expression groups. Kaplan Meier analysis showed that high expression of *MAP3K14* was significantly correlated with poorer OS in HCC patients (*p*=0.025, Figure 2D). In addition, the Kaplan Meier Plotter database was used to evaluate the prognostic significance of *MAP3K14* for HCC. The prognosis data showed that the progression free survival (PFS) (*p*=0.0053, Figure 2E) and recurrence free survival (RFS) (*p*=0.015, Figure 2F) were shorter in HCC patients with higher *MAP3K14* expression, while patients with lower *MAP3K14* expression had better survival outcomes.

### 3.3 External validation of protein expression of MAP3K14 and its relationship to prognosis in patients with HCC

To validate our results at the protein level, we performed IHC staining to detect the expression of MAP3K14 in 180 HCC tissue samples (including 94 tumor samples and 86 normal tissue samples). The immunostaining was mainly localized in the cytoplasm of HCC cells. As shown in Figure 2G and H, according to the cytoplasmic staining score criteria, the protein level of MAP3K14 was significantly higher in liver cancer tissues compared to the corresponding non-cancerous tissues, which is consistent with the predicted results from public databases. We further evaluated the prognostic significance of *MAP3K14* in HCC using survival analysis. The results indicated that patients with higher expression levels of MAP3K14 had shorter overall survival (OS), suggesting that *MAP3K14* may be a risk factor for HCC development (Figure 2I).

### 3.4 Association between MAP3K14 expression and clinical characteristics

As shown in Table 1, the association between *MAP3K14* expression and clinical features was analyzed using chi-square test or Fisher's exact test. High expression of *MAP3K14* in HCC was associated with higher Pathologic T stage (*p*=0.026) and Pathologic stage (*p*=0.032). There were more patients with tumors in the high expression group (*p*=0.024), but they exhibited lower levels of AFP (*p*=0.002). However, there was no significant correlation between *MAP3K14* expression and clinical features such as age, race, M stage, and N stage.

### 3.5 Cox Regression Model Analysis

As shown in Table 2, univariate and multivariate Cox regression analyses were performed based on TCGA-LIHC data to investigate potential risk factors affecting the prognosis of HCC patients. In univariate Cox regression analysis, pathological stage (HR=2.46; *p* < 0.001) and *MAP3K14* (HR=1.48; *p*=0.026) were associated with overall survival of HCC patients. Multivariate Cox regression analysis, which further incorporated statistically significant variables from univariate analysis, showed that pathological stage (HR=2.44; *p* < 0.001) and *MAP3K14* (HR=1.44; *p*=0.048) were independent prognostic factors for OS in HCC patients. It is suggested that the expression level of *MAP3K14* is associated with the prognosis of HCC patients.

### 3.6 MAP3K14-Associated PPI Network

**Figure 3:**
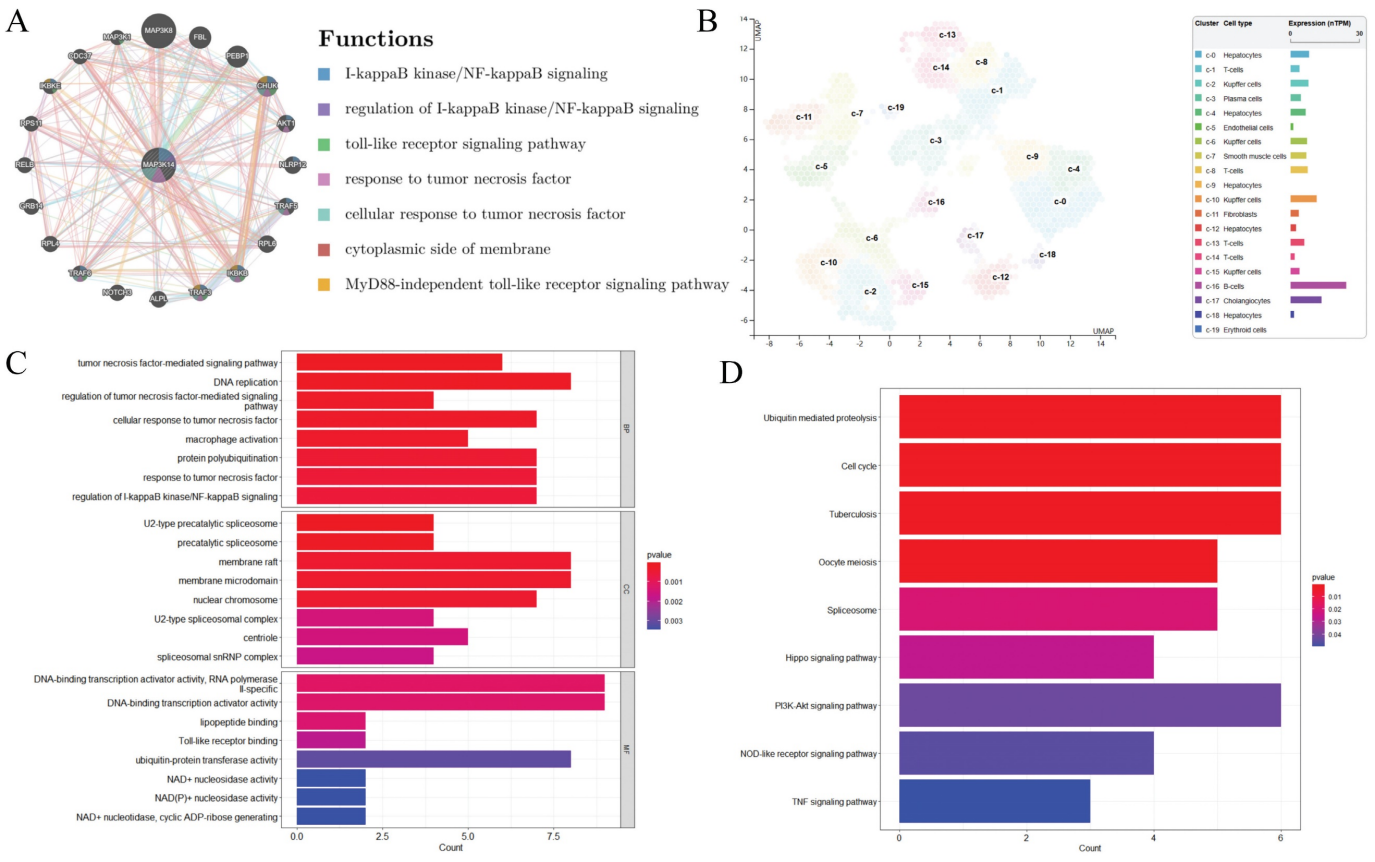
Signaling pathways and biological processes of *MAP3K14* (A) Protein network diagram of interaction with *MAP3K14* protein. (B) RNA levels of *MAP3K14* and marker genes in different single-cell type clusters of liver. (C-D) GO analysis and KEGG pathway reveal the underlying mechanism of *MAP3K14* in the promotion of HCC. GO: Gene Ontology; KEGG: Kyoto Encyclopedia of Genes and Genomes.

The GeneMANIA database has identified a total of 20 proteins that are known or predicted to interact with *MAP3K14*, including MAP3K8, FBL, PEBP1, CHUK, AKT1, NLRP12, TRAF5, RPL6, IKBKB, TRAF3, etc. Nodes with different colors represent different interaction relationships (Figure 3A). The interactions are associated with various biological processes, including I-kappaB kinase/NF-kappaB signaling, regulation of I-kappaB kinase/NF-kappaB signaling, toll-like receptor signaling pathway, response to tumor necrosis factor, cellular response to tumor necrosis factor, cytoplasmic side of membrane, and MyD88-independent toll-like receptor signaling pathway.

### 3.7 MAP3K14 Expression in Different Cells of HCC

Single cell data from the HPA database were visualized using UMAP. Each color represents a single cell cluster identified after clustering analysis and each scatter represents a cell that was divided into a total of 19 cell populations in the liver. The results showed that *MAP3K14* mRNA levels were higher in B cells and bile duct cells and lower in red lineage cells (Figure 3B, C).

### 3.8 MAP3K14‐Related Genes and Functional Enrichment Analysis

To explore the possible biological functions of *MAP3K14* in the pathogenesis and progression of HCC, the top 200 genes highly associated with *MAP3K14* searched in the GEPIA database were used. Next, these genes were used for GO and KEGG enrichment analysis and functional annotation tables were obtained. As shown in Figure 3D, In GO Biological Processes, the above genes were mainly involved in tumor necrosis factor-mediated signaling pathway, DNA replication, regulation of tumor necrosis factor-mediated signaling pathway, cellular response to tumor necrosis factor, and macrophage activation. In Cellular components, these genes were closely associated with U2-type precatalytic spliceosome, membrane raft, membrane microdomain, and nuclear chromosome. Molecular functions were primarily co-regulated with DNA-binding transcription activator activity, RNA polymerase II-specific, lipopeptide binding, Toll-like receptor binding, and ubiquitin-protein transferase activity. Additionally, in KEGG analysis, 9 signaling pathways finally showed statistical significance, including in Ubiquitin mediated proteolysis, Cell cycle, PI3K-Akt signaling pathway, Hippo signaling pathway, NOD-like receptor signaling pathway and TNF signaling pathway (Figure 3E).

### 3.9 Correlation Analysis between MAP3K14 Expression and Tumor‐Infiltrating Immune Cells in HCC

**Figure 4:**
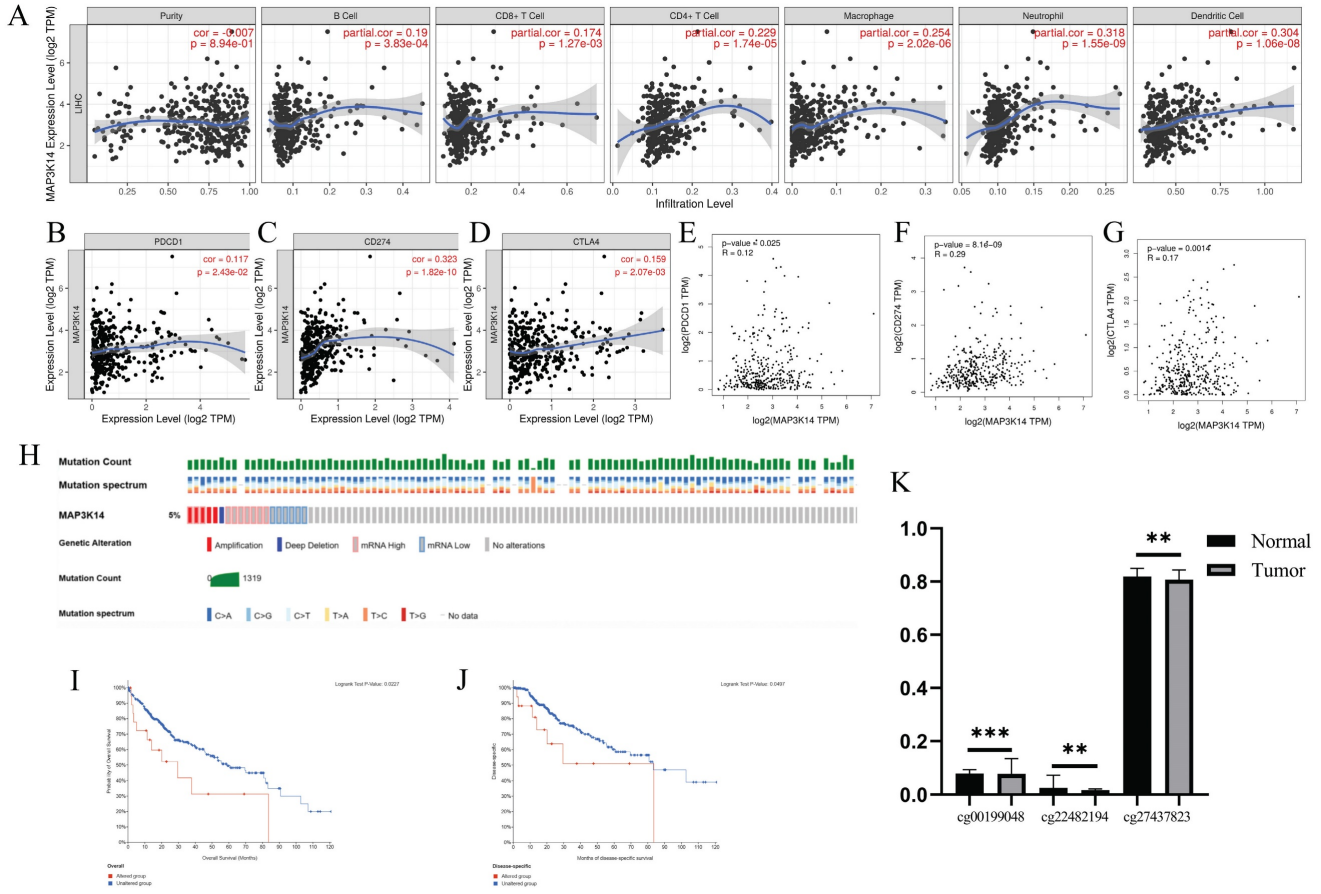
The relationship of immune cell infiltration with *MAP3K14* level in HCC. (A) The correlation of *MAP3K14* expression level with B cell, CD8+ T cell, CD4+ T cell, macrophage, neutrophil, and dendritic cell infiltration level in HCC. (B-D) The correlation of *MAP3K14* with the expression of PD1, PD-L1 and CTLA-4 in HCC was determined by the TIMER database. (E-G) The correlation of *MAP3K14* with the expression of PD1, PD-L1 and CTLA-4 in HCC was determined by the GEPIA database. (H) Onco-Print plot of *MAP3K14* alterations. (I-J) Genetic alterations in *MAP3K14* are associated with shorter OS and DSS in HCC patients. (K) The methylation levels of the *MAP3K14* promoter region in tumor tissues and normal tissues. DSS: Disease Specific Survival.

Immune cell infiltration plays a crucial role in tumors. Therefore, the correlation between *MAP3K14* expression levels and immune cell infiltration levels was assessed. Analysis of the "gene" module showed the correlation between *MAP3K14* expression levels and the level of immune cell infiltration. As shown in Figure 4A, we found a significant positive correlation between *MAP3K14* expression level and B cells, CD8^+^ T cells, CD4^+^ T cells, macrophages, neutrophils, and dendritic cells. These data suggest that the potential regulatory mechanism of *MAP3K14* in HCC may function by influencing immune cell infiltration.

### 3.10 Relationship between MAP3K14 expression levels and immune checkpoints in hepatocellular carcinoma

PD1/PD-L1 and CTLA-4 are important immune checkpoints responsible for tumor immune escape. Considering the potential oncogenic role of *MAP3K14* in HCC, the relationship of *MAP3K14* with PD1, PD-L1 or CTLA-4 was evaluated. *MAP3K14* expression was significantly and positively correlated with PD1, PD-L1 and CTLA-4 in HCC, adjusted by purity (Figure 4B-D). Like the TIMER database analysis, in HCC, *MAP3K14* was significantly positively associated with PD1, PD-L1, or CTLA-4 (Figure 4E-G). These results suggest that *MAP3K14*-mediated HCC may be associated with tumor immune evasion.

### 3.11 MAP3K14 mutation landscape in hepatocellular carcinoma

The occurrence and progression of malignancies are influenced by mutations in specific regulatory genes. cBioportal database contains several databases and provides visualization tools for the analysis of cancer-related genetic data. The type and frequency of *MAP3K14* mutations in HCC were investigated according to data filtering and extraction conditions. A dataset containing 372 samples with information on genetic alterations (TCGA, Pan-Cancer Atlas) was selected from the cBioPortal database for analysis. The prognostic value of *MAP3K14* gene alterations in HCC was further investigated (Figure 4H). The results showed that about 5% of HCC patients had *MAP3K14* gene alterations (Figure 4H). The prognostic results showed that HCC patients with *MAP3K14* gene alterations had lower OS and DSS than those without gene alterations (Figure 4I, J).

### 3.12 The correlation between mRNA expression and methylation of MAP3K14

Under normal circumstances, high methylation levels in gene promoters are associated with gene silencing or suppression. We downloaded the methylation value matrix of HCC samples from the UCSC Xena database and retrieved 20 methylation sites in the promoter region of *MAP3K14* from the MethSurv database. As shown in Figure 4K, through a comparison between HCC tissues and normal tissues, we found that the methylation levels in three promoter regions (cg00199048, cg22482194, and cg27437823) were significantly lower in the tumor group than in the normal group. Generally, the methylation level in the promoter region is negatively correlated with gene expression, which partially explains the upregulation of *MAP3K14* in tumor tissues.

### 3.13 Correlation between MAP3K14 Expression and Drug Sensitivity

**Figure 5:**
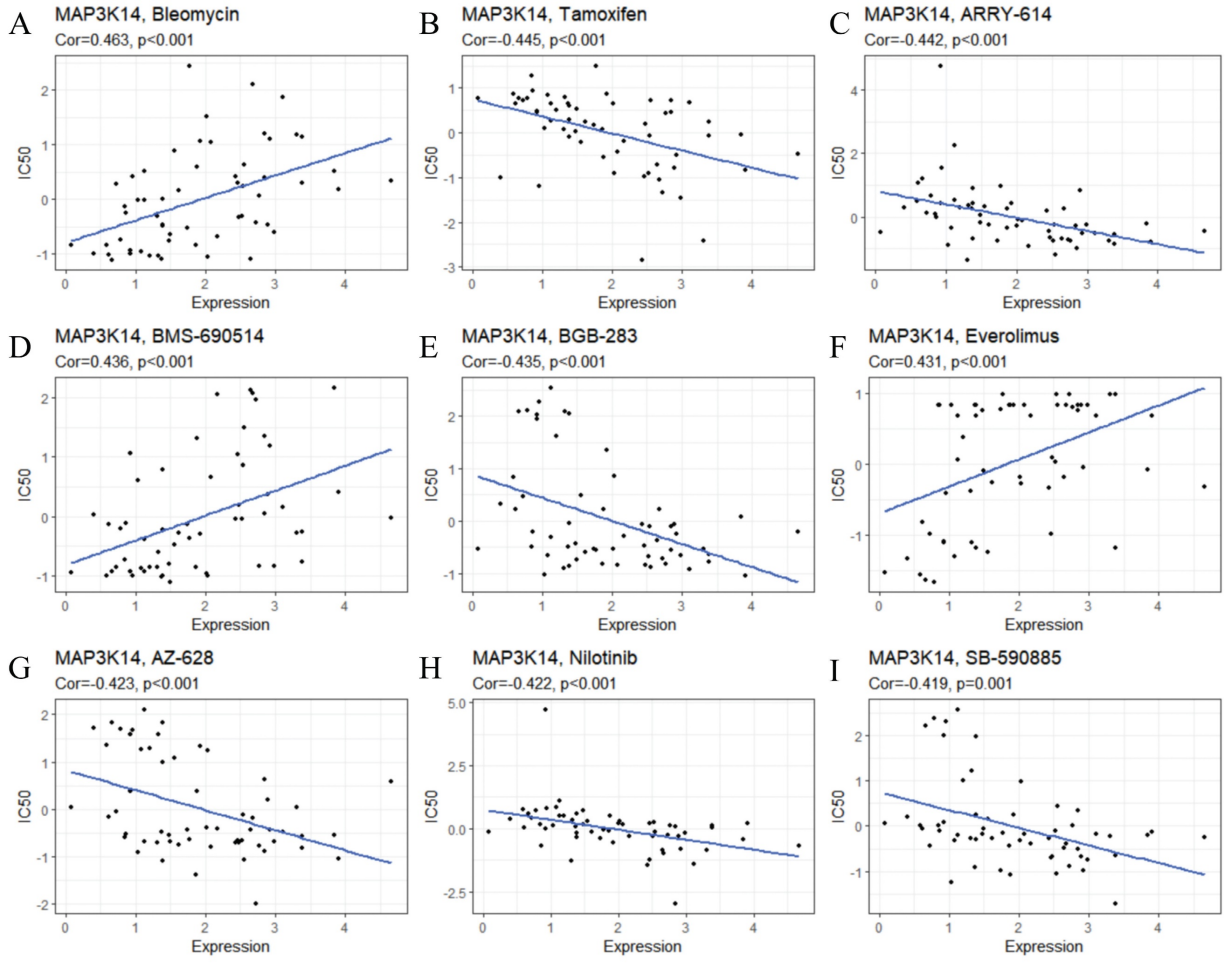
The relationship between *MAP3K14* expression and the response to 9 different chemotherapy drugs.

To ensure the reliability of the analysis results, we selected drugs that have been clinically tested and FDA‐approved for drug sensitivity analysis. In Figure 5A-I, the results showed that *MAP3K14* expression was significantly positively correlated with Bleomycin, BMS-690514, and Everolimus. The sensitivity of *MAP3K14* to Tamoxifen, Pexmetinib (ARY-614), Lifirafenib (BGB-283), AZ-628, Nilotinib and SB-590885 increased with the increase of *MAP3K14* expression. Given these findings, *MAP3K14* may become a novel marker for targeted therapies for HCC.

### 3.14 Prediction and analysis of upstream miRNAs of MAP3K14

**Figure 6:**
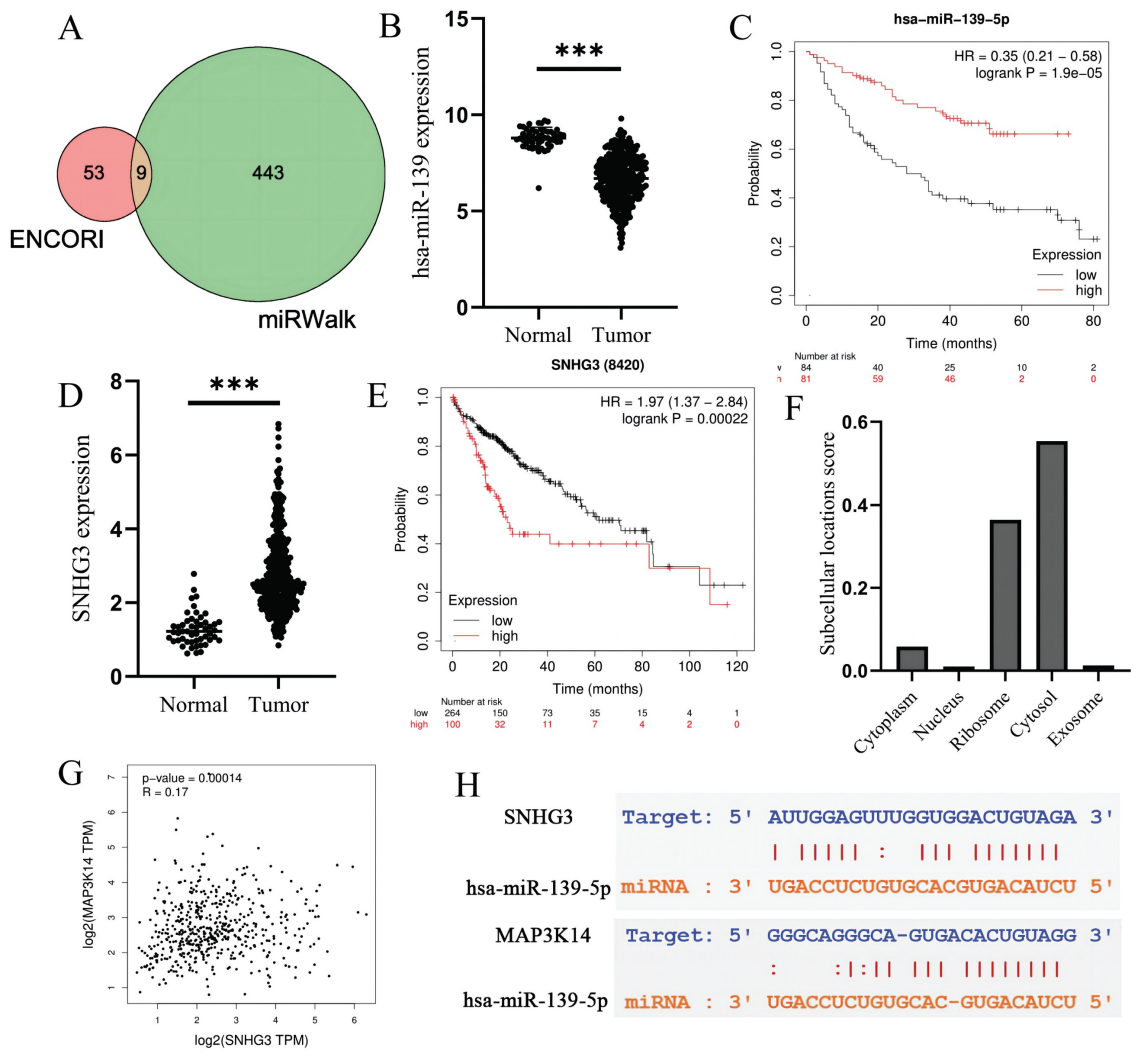
Prediction of potential ceRNA network. **(A)** *MAP3K14* target miRNA prediction based on ENCORI and miRWalk databases. **(B)** The expression status of has-miR-139-5p in HCC tissues and normal tissues. **(C)** The relationship between the expression of *hsa-miR-139-5p* and overall survival (OS) in patients with liver cancer. **(D)** The expression status of *SNHG3* in HCC tissues and normal tissues. **(E)** The relationship between the expression of *SNHG3* and overall survival (OS) in patients with liver cancer. **(F)** The subcellular localization prediction of *SNHG3*. **(G)** The relationship between *SNHG3* and *MAP3K14* expression by GEPIA database. **(G)** The target sites in the *SNHG3* and *MAP3K14* 3' UTRs were predicted to pair with *hsa-miR-139-5p* by ENCORI database. *SNHG3*: Small Nucleolar RNA Host Gene 3.

It has been widely acknowledged that ncRNAs are responsible for the regulation of gene expression. To ascertain whether *MAP3K14* was modulated by some ncRNAs, we first predicted upstream miRNAs that could potentially bind to *MAP3K14* and finally found 9 miRNAs (Figure 6A). Based on the action mechanism of miRNA in regulation of target gene expression, there should be negative correlation between miRNA and *MAP3K14*. The expression level of *hsa-miR-139-5p* was significantly lower in normal tissues than in tumor tissues (Figure 6B). Finally, the prognostic value of *hsa-miR-139-5p* in HCC was determined. *hsa-miR-139-5p* is significantly down-regulated in HCC, and it's up-regulation is positively correlated with patient prognosis (Figure 6C). All these findings suggest that *hsa-miR-139-5p* might be the most potential regulatory miRNA of *MAP3K14* in HCC.

### 3.15 Prediction and analysis of upstream lncRNAs of hsa-miR-139-5p

Next, the upstream lncRNAs of *hsa-miR-139-5p* were predicted using ENCORI database. A total of 20 possible lncRNAs were forecasted. Then, the expression level and prognostic value of these lncRNAs in HCC were detected. Among these 20 lncRNAs, AC124798.1 and *SNHG3* were significantly up-regulated in HCC compared with normal controls, and the high-expression group had a worse prognosis. Then through Lnclocator database to predict the two lncRNA subcellular localization, only *SNHG3* mainly locate in the cytosol (Figure 6F). Figure 6D shows the difference in expression of *SNHG3* in normal and tumor tissues, and the prognosis of high expression of *SNHG3* is worse (Figure 6E). According to the competing endogenous RNA (ceRNA) hypothesis, lncRNA could increase mRNA expression by competitively binding to shared miRNAs. Therefore, there should be positive correlation between lncRNA and mRNA. Finally, we conducted correlation analysis on the expression of *SNHG3* and *MAP3K14*. As shown in Figure 6G, *SNHG3* was significantly positively correlated with *MAP3K14*. The target sites in the *SNHG3* and *MAP3K14* 3'UTRs were predicted to pair with *hsa-miR-139-5p* by ENCORI database (Figure 6H). Based on differential expression analysis, survival analysis and correlation analysis, *SNHG3* might be the most potential upstream lncRNAs of *hsa-miR-139-5p*/*MAP3K14* axis in HCC.

## 4. Discussion

HCC is one of the leading causes of cancer mortality worldwide because of its poor prognosis and high aggressiveness 28. Several biomarkers associated with HCC have been discovered in recent years. Elucidating the molecular mechanisms of HCC occurrence will provide important clues for developing effective therapeutic targets or finding promising prognostic markers. *MAP3K14*, also known as NF-κB-inducible kinase or NIK. According to reports, NIK (NF-κB-inducing kinase) plays a crucial role in various diseases. When NIK is excessively active, it may be associated with diseases such as autoimmune disorders, sterile inflammation, fibrosis, and cancer 29. Previous studies have shown that changes in NIK gene or protein expression occur in hematological tumors such as multiple myeloma 30, leukemia 31, and melanoma 32, and that targeting NIK can reduce tumor cell survival. At the same time, the importance of NIK is also shown in solid tumors. Studies have shown that NIK expression is significantly increased in tumor tissues of breast cancer patients, which may be an important factor affecting the prognosis of breast cancer patients 33. In the progression of non-alcoholic steatohepatitis (NASH), NASH-related hepatocellular carcinoma (HCC), and liver cancer, NIK and JAK2/STAT5 signaling in the liver play a significant role. Inhibiting hepatic NIK may lead to increased sensitivity of STAT5, thus serving as a potential strategy for treating NASH and preventing its progression to liver cancer 34. Furthermore, studies have shown that a specific allele of the MAP3K14 gene is associated with poorer survival rates in patients with hepatitis B virus-related hepatocellular carcinoma (HBV-HCC), and this allele is significantly correlated with elevated expression levels of MAP3K14 mRNA in liver tissue 35. This provides new insights for prognosis assessment and drug development in HBV-HCC patients. Additionally, research has revealed upregulation of NIK expression in HCC tissues and cells. Knockdown of NIK reduces the cancer stem cell-like characteristics of HCC cells 36. These findings offer important clues for further understanding the role of NIK in disease progression and developing relevant therapeutic approaches. However, further elucidation is still needed regarding the association between MAP3K14 expression and hepatocellular carcinoma (HCC), as well as its role in HCC.

In this study, we first analyzed the pan-cancer expression of *MAP3K14* using the TIMER2.0 database and found that *MAP3K14* is highly expressed in various cancers, including HCC. Based on data from the TCGA database, we confirmed the high expression of *MAP3K14* in HCC. We further validated the expression of *MAP3K14* using the UALCAN database and GEO datasets of paired HCC samples, which showed significantly higher expression levels of *MAP3K14* in tumor tissues compared to adjacent non-tumor tissues. We analyzed the role of *MAP3K14* in HCC using the UALCAN database and found that *MAP3K14* is closely associated with individual cancer stage, tumor grade, and age in HCC. Survival analysis revealed a correlation between elevated *MAP3K14* expression and lower survival rates in HCC patients, with significantly lower overall survival (OS), progression-free survival (PFS), and recurrence-free survival (RFS) in patients with high *MAP3K14* expression compared to those with low expression. We further validated our results at the protein level through immunohistochemical staining and assessed the prognostic significance of *MAP3K14* in HCC using survival analysis. The results showed that *MAP3K14* protein levels were significantly higher in liver cancer tissues compared to corresponding non-cancerous tissues, and patients with higher *MAP3K14* expression had shorter overall survival, consistent with the analysis of public databases. This suggests a potential correlation between *MAP3K14* and the occurrence and development of HCC. Furthermore, univariate, and multivariate Cox regression analysis demonstrated that *MAP3K14* is an independent factor influencing the prognosis of HCC patients.

Next, we investigated the potential biological functions of *MAP3K14* in HCC.GO and KEGG pathway analysis revealed a variety of signaling pathways, such as PI3K-Akt signaling pathway, NOD-like receptor signaling pathway, and TNF signaling pathway. The PI3K-AKT pathway is a key regulatory center that regulates cell growth, metabolism, proliferation, survival, transcription, and protein synthesis. It has been reported that the activation of PI3K-AKT pathway is related to the progression and metastasis of HCC 37, 38. Currently, the PI3K-AKT pathway has become a focal point of attention in tumor progression. Inhibitors targeting this pathway have brought new prospects for targeted cancer therapy.

Many studies have demonstrated that tumor immune cell infiltration can influence the efficacy of chemotherapy, radiotherapy or immunotherapy and the prognosis of cancer patients 39, 40. Our work showed that *MAP3K14* was significantly and positively associated with various immune cells, including B cells, CD8^+^ T cells, CD4^+^ T cells, macrophages, neutrophils, and dendritic cells in HCC. This implies that *MAP3K14* may influence the interaction between immune cells and malignant tumor cells, thereby regulating the progression of HCC. These data suggest that the potential regulatory mechanism of *MAP3K14* in HCC may function by influencing immune cell infiltration. Immunotherapy has emerged as a promising therapy for the treatment of several solid tumors and various immune checkpoint molecules such as PD-1/PD-L1 and CTLA-4 have been found to play an important role in tumor immune escape 41. Immune checkpoints play a key role in regulating immune cell function and tumor immune cell infiltration, and in recent years ICI therapies have revolutionized the treatment of advanced malignancies and other diseases 42, ICIs targeting PD-1, PD-L1 or CTLA 4 enable long-term survival of patients with tumors 43, 44. Therefore, we also evaluated the relationship between *MAP3K14* and immune checkpoints. The results showed that *MAP3K14* correlated with the expression of PD-1, PD-L1 and CTLA 4, implying a possible role in regulating immune escape.

The etiology of HCC is closely linked to environmental factors, and epigenetic aberrations may contribute to the development and promotion of HCC 45. Epigenetic modifications and alterations dysregulate the expression of tumor suppressor genes and oncogenes, leading to carcinogenesis, progression, and metastasis of HCC 46, 47. By using the MethSurv database, a total of 20 methylated CpG sites of *MAP3K14* were identified, we further explored the potential mechanism of *MAP3K14* up-regulation in HCC and found that DNA hypomethylation in the promoter region of *MAP3K14* may lead to its increased expression in HCC. Genetic alterations and CNVs are common sources of dysregulated gene expression 48, we further explored the relationship between the mutation status of *MAP3K14* in hepatocellular carcinoma samples and prognosis. Prognostic results showed that HCC patients with *MAP3K14* gene alterations had significantly lower OS and DSS than those without gene alterations.

The ceRNA regulatory network has been reported to be involved in the development and progression of many human cancers, including lung cancer 49, gastric cancer 50 and colorectal cancer 51. ncRNAs are involved in the regulation of gene expression through the ceRNA mechanism. In this study, we attempted to establish a *MAP3K14*-associated ceRNA triplex network in HCC, as well as to correlate it with prognosis. To explore the upstream regulatory miRNAs of *MAP3K14*, we first predicted 9 miRNAs that might bind to *MAP3K14* by database. miRNAs were further analyzed for their expression and prognostic value in HCC, and *hsa-miR-139-5p* might be the most promising regulatory miRNA for *MAP3K14* in HCC. According to many previous reports, miR-139 can act as an oncogene regulator in cancer, and LncRNA XIST can promote HCC growth by regulating miR-139-5p 52. Next, the upstream lncRNAs of the *hsa-miR-139-5p*/*MAP3K14* axis were predicted and 20 possible lncRNAs were identified. by performing expression analysis, survival analysis and correlation analysis, *SNHG3* was identified as the most likely up-regulated lncRNA. *SNHG3* has been reported to play a carcinogenic role in the development of HCC 53, and *SNHG3* is up-regulated in HCC tissues and cells, while miR-139-5p is down-regulated. In addition, *SNHG3* knockdown or miR-139-5p overexpression could inhibit the proliferation, migration, and invasion of HCC cells. In conclusion, the *SNHG3*/*hsa-miR-139-5p*/*MAP3K14* axis was identified as a possible potential regulatory pathway in HCC.

In addition, by targeted drug sensitivity analysis, we found that the expression of *MAP3K14* was negatively correlated with the sensitivity to a variety of targeted drugs, suggesting that *MAP3K14* may be a potential therapeutic target for HCC. Down-regulation of the expression of *MAP3K14* may improve the sensitivity of targeted drug therapy for hepatocellular carcinoma and improve the prognosis of patients.

## 5. Conclusions

In conclusion, the present study demonstrates that *MAP3K14* is highly expressed in HCC and is associated with unfavorable prognosis in HCC. It may be involved in the development of hepatocellular carcinoma by regulating immune cell infiltration and may serve as a marker for targeted therapy in hepatocellular carcinoma patients. However, several limitations of this study need to be discussed. First, we verified the high expression of *MAP3K14* in tumor tissues and poor prognosis in TCGA and GEO cohorts and immunohistochemistry. However, this study is only a retrospective study, and further prospective studies with a large enough sample size are needed to confirm each other. In addition, this study failed to collect all clinical information, which may affect the prognosis of HCC patients. Secondly, we constructed the *lnc-SNHG3*/*hsa-miR-139-5p*/*MAP3K14* overexpression ceRNA network related to HCC prognosis, and further experimental studies are needed to investigate the role and mechanism of the* lnc-SNHG3*/*MAP3K14* axis in HCC.

## Figures and Tables

**Table 1:** The relationship between *MAP3K14* expression and clinical characteristics in HCC.

Characteristics	Low expression of *MAP3K14*	High expression of *MAP3K14*	*P* value
n	187	187	
Gender, n (%)			0.060
Female	69 (18.4%)	52 (13.9%)	
Male	118 (31.6%)	135 (36.1%)	
Age, n (%)			0.275
<= 60	94 (25.1%)	83 (22.2%)	
> 60	93 (24.9%)	103 (27.5%)	
Missing	0 (0%)	1 (0.3%)	
Pathologic T stage, n (%)			**0.026**
T1	102 (27.3%)	81 (21.7%)	
T3&T4&T2	83 (22.2%)	105 (28.0%)	
Missing	2 (0.5%)	1 (0.3%)	
Pathologic N stage, n (%)			0.681
N0	132 (35.3%)	122 (32.6%)	
N1	3 (0.8%)	1 (0.3%)	
Missing	52 (13.9%)	64 (17.1%)	
Pathologic M stage, n (%)			0.176
M0	143 (38.2%)	125 (33.4%)	
M1	4 (1.1%)	0 (0%)	
Missing	40 (10.7%)	62 (16.6%)	
Pathologic stage, n (%)			**0.032**
Stage I	98 (26.2%)	75 (20.1%)	
Stage II&Stage III&Stage IV	80 (21.4%)	97 (25.9%)	
Missing	9 (2.4%)	15 (4.0%)	
Tumor status, n (%)			**0.024**
Tumor free	109 (29.2%)	93 (24.9%)	
With tumor	64 (17.1%)	89 (23.8%)	
Missing	14 (3.7%)	5 (1.3%)	
Race, n (%)			0.249
Black or African American&White	95 (25.4%)	107 (28.6%)	
Asian	85 (22.7%)	75 (20.1%)	
Missing	7 (1.9%)	5 (1.3%)	
Histologic grade, n (%)			0.867
G1	28 (7.5%)	27 (7.2%)	
G3&G4&G2	156 (41.7%)	158 (42.3%)	
Missing	3 (0.8%)	2 (0.5%)	
AFP (ng/ml), n (%)			**0.002**
<= 400	99 (26.4%)	116 (31.0%)	
> 400	44 (11.8%)	21 (5.6%)	
Missing	44 (11.8%)	50 (13.4%)	
Child-Pugh grade, n (%)			0.246
A	111 (29.7%)	108 (28.9%)	
B&C	14 (3.7%)	8 (2.1%)	
Missing	62 (16.6%)	71 (19.0%)	
Albumin(g/dl), n (%)			0.167
< 3.5	40 (10.7%)	29 (7.8%)	
>= 3.5	112 (29.9%)	119 (31.8%)	
Missing	35 (9.4%)	39 (10.4%)	
Vascular invasion, n (%)			0.764
No	106 (28.3%)	102 (27.3%)	
Yes	58 (15.5%)	52 (13.9%)	
Missing	23 (6.2%)	33 (8.8%)	
Prothrombin time, n (%)			0.586
<= 4	105 (35.4%)	103 (27.5%)	
> 4	48 (16.2%)	41 (11.0%)	
Missing	34 (9.1%)	43 (11.5%)	

**Table 2:** Univariate and multivariate Cox regression analysis of clinicopathologic characteristics associated with OS in the TCGA sample.

Variable	Univariable		Multivariable	
	HR (95% CI)	*P*	HR (95% CI)	*P*
Age (>60 *vs.* ≤60)	1.22 (0.864-1.733)	0.256		
Child-Pugh grade (B&C *vs.* A)	1.64 (0.811-3.330)	0.168		
AFP (ng/ml) (>400 *vs.* ≤400)	1.06 (0.649-1.731)	0.817		
Histologic grade (G3&G4 *vs.* G1&G2)	1.11 (0.778-1.592)	0.557		
Prothrombin time (>4 *vs.* ≤4)	1.31 (0.867-1.984)	0.200		
relative family cancer history (Yes *vs.* No)	1.19 (0.825-1.716)	0.352		
Gender (Male *vs.* Female)	1.25 (0.876-1.776)	0.220		
Race (Non-Asian *vs.* Asian)	1.38 (0.959-1.992)	0.083		
Pathologic stage (StageⅢ&Ⅳ *vs.* StageⅠ&Ⅱ)	2.46 (1.701-3.568)	**<0.001**	2.44 (1.684-3.536)	**<0.001**
BMI (>25 *vs.* ≤25)	0.82 (0.563-1.181)	0.280		
*MAP3K14* (High *vs.* Low)	1.48 (1.049-2.086)	**0.026**	1.44 (1.003-2.077)	**0.048**

## References

[1] Sung H, Ferlay J, Siegel RL, Laversanne M, Soerjomataram I, Jemal A (2021). Global Cancer Statistics 2020: GLOBOCAN Estimates of Incidence and Mortality Worldwide for 36 Cancers in 185 Countries. CA Cancer J Clin.

[2] Yang JD, Hainaut P, Gores GJ, Amadou A, Plymoth A, Roberts LR (2019). A global view of hepatocellular carcinoma: trends, risk, prevention and management. Nat Rev Gastroenterol Hepatol.

[3] Singal AG, Lampertico P, Nahon P (2020). Epidemiology and surveillance for hepatocellular carcinoma: New trends. J Hepatol.

[4] Craig AJ, von Felden J, Garcia-Lezana T, Sarcognato S, Villanueva A (2020). Tumour evolution in hepatocellular carcinoma. Nat Rev Gastroenterol Hepatol.

[5] Huang A, Yang XR, Chung WY, Dennison AR, Zhou J (2020). Targeted therapy for hepatocellular carcinoma. Signal Transduct Target Ther.

[6] Tabrizian P, Holzner ML, Mehta N, Halazun K, Agopian VG, Yao F (2022). Ten-Year Outcomes of Liver Transplant and Downstaging for Hepatocellular Carcinoma. JAMA Surg.

[7] Carroll HK, Duffy AG, O'Farrelly C (2022). Liver Immunology, Immunotherapy, and Liver Cancers: Time for a Rethink?. Semin Liver Dis.

[8] Harhaj EW, Dixit VM (2011). Deubiquitinases in the regulation of NF-κB signaling. Cell Res.

[9] Xiao G, Harhaj EW, Sun SC (2001). NF-kappaB-inducing kinase regulates the processing of NF-kappaB2 p100. Mol Cell.

[10] Pflug KM, Sitcheran R (2020). Targeting NF-κB-Inducing Kinase (NIK) in Immunity, Inflammation, and Cancer. Int J Mol Sci.

[11] Maubach G, Feige MH, Lim MCC, Naumann M (2019). NF-kappaB-inducing kinase in cancer. Biochim Biophys Acta Rev Cancer.

[12] Hayashi Y, Nakayama J, Yamamoto M, Maekawa M, Watanabe S, Higashiyama S (2023). Aberrant accumulation of NIK promotes tumor growth by dysregulating translation and post-translational modifications in breast cancer. Cancer Cell Int.

[13] Goldman MJ, Craft B, Hastie M, Repečka K, McDade F, Kamath A (2020). Visualizing and interpreting cancer genomics data via the Xena platform. Nat Biotechnol.

[14] Barrett T, Wilhite SE, Ledoux P, Evangelista C, Kim IF, Tomashevsky M (2013). NCBI GEO: archive for functional genomics data sets-update. Nucleic Acids Res.

[15] Chandrashekar DS, Karthikeyan SK, Korla PK, Patel H, Shovon AR, Athar M (2022). UALCAN: An update to the integrated cancer data analysis platform. Neoplasia.

[16] Győrffy B (2021). Survival analysis across the entire transcriptome identifies biomarkers with the highest prognostic power in breast cancer. Comput Struct Biotechnol J.

[17] Warde-Farley D, Donaldson SL, Comes O, Zuberi K, Badrawi R, Chao P (2010). The GeneMANIA prediction server: biological network integration for gene prioritization and predicting gene function. Nucleic Acids Res.

[18] Asplund A, Edqvist PH, Schwenk JM, Pontén F (2012). Antibodies for profiling the human proteome-The Human Protein Atlas as a resource for cancer research. Proteomics.

[19] Tang Z, Li C, Kang B, Gao G, Li C, Zhang Z (2017). GEPIA: a web server for cancer and normal gene expression profiling and interactive analyses. Nucleic Acids Res.

[20] Li T, Fu J, Zeng Z, Cohen D, Li J, Chen Q (2020). TIMER2.0 for analysis of tumor-infiltrating immune cells. Nucleic Acids Res.

[21] Gao J, Aksoy BA, Dogrusoz U, Dresdner G, Gross B, Sumer SO (2013). Integrative analysis of complex cancer genomics and clinical profiles using the cBioPortal. Sci Signal.

[22] Ding W, Chen J, Feng G, Chen G, Wu J, Guo Y (2020). DNMIVD: DNA methylation interactive visualization database. Nucleic Acids Res.

[23] Modhukur V, Iljasenko T, Metsalu T, Lokk K, Laisk-Podar T, Vilo J (2018). MethSurv: a web tool to perform multivariable survival analysis using DNA methylation data. Epigenomics.

[24] Sticht C, De La Torre C, Parveen A, Gretz N (2018). miRWalk: An online resource for prediction of microRNA binding sites. PLoS One.

[25] Li JH, Liu S, Zhou H, Qu LH, Yang JH (2014). starBase v2.0: decoding miRNA-ceRNA, miRNA-ncRNA and protein-RNA interaction networks from large-scale CLIP-Seq data. Nucleic Acids Res.

[26] Cao Z, Pan X, Yang Y, Huang Y, Shen HB (2018). The lncLocator: a subcellular localization predictor for long non-coding RNAs based on a stacked ensemble classifier. Bioinformatics.

[27] Reinhold WC, Sunshine M, Liu H, Varma S, Kohn KW, Morris J (2012). CellMiner: a web-based suite of genomic and pharmacologic tools to explore transcript and drug patterns in the NCI-60 cell line set. Cancer Res.

[28] Jasirwan COM, Hasan I, Sulaiman AS, Lesmana CRA, Kurniawan J, Kalista KF (2020). Risk factors of mortality in the patients with hepatocellular carcinoma: A multicenter study in Indonesia. Curr Probl Cancer.

[29] Valiño-Rivas L, Vaquero JJ, Sucunza D, Gutierrez S, Sanz AB, Fresno M (2019). NIK as a Druggable Mediator of Tissue Injury. Trends Mol Med.

[30] Annunziata CM, Davis RE, Demchenko Y, Bellamy W, Gabrea A, Zhan F (2007). Frequent engagement of the classical and alternative NF-kappaB pathways by diverse genetic abnormalities in multiple myeloma. Cancer Cell.

[31] Saitoh Y, Yamamoto N, Dewan MZ, Sugimoto H, Martinez Bruyn VJ, Iwasaki Y (2008). Overexpressed NF-kappaB-inducing kinase contributes to the tumorigenesis of adult T-cell leukemia and Hodgkin Reed-Sternberg cells. Blood.

[32] Thu YM, Su Y, Yang J, Splittgerber R, Na S, Boyd A (2012). NF-κB inducing kinase (NIK) modulates melanoma tumorigenesis by regulating expression of pro-survival factors through the β-catenin pathway. Oncogene.

[33] Zhang X, Wang Y, Mao Z, Huang D, Zhou J, Wang X (2015). Expression of NF-κB-inducing kinase in breast carcinoma tissue and its clinical significance. Int J Clin Exp Pathol.

[34] Vesting AJ, Jais A, Klemm P, Steuernagel L, Wienand P, Fog-Tonnesen M (2022). NIK/MAP3K14 in hepatocytes orchestrates NASH to hepatocellular carcinoma progression via JAK2/STAT5 inhibition. Mol Metab.

[35] Huang Q, Liu Y, Qiu M, Lin Q, Wei X, Zhou Z (2022). Potentially functional variants of MAP3K14 in the NF-κB signaling pathway genes predict survival of HBV-related hepatocellular carcinoma patients. Front Oncol.

[36] Daren L, Dan Y, Jinhong W, Chao L (2024). NIK-mediated reactivation of SIX2 enhanced the CSC-like traits of hepatocellular carcinoma cells through suppressing ubiquitin-proteasome system. Environ Toxicol.

[37] Rahmani F, Ziaeemehr A, Shahidsales S, Gharib M, Khazaei M, Ferns GA (2020). Role of regulatory miRNAs of the PI3K/AKT/mTOR signaling in the pathogenesis of hepatocellular carcinoma. J Cell Physiol.

[38] Brown JS, Banerji U (2017). Maximising the potential of AKT inhibitors as anti-cancer treatments. Pharmacol Ther.

[39] Batista A, Rodvold JJ, Xian S, Searles SC, Lew A, Iwawaki T (2020). IRE1α regulates macrophage polarization, PD-L1 expression, and tumor survival. PLoS Biol.

[40] Zhang Y, Zhang Z (2020). The history and advances in cancer immunotherapy: understanding the characteristics of tumor-infiltrating immune cells and their therapeutic implications. Cell Mol Immunol.

[41] Chang H, Jung W, Kim A, Kim HK, Kim WB, Kim JH (2017). Expression and prognostic significance of programmed death protein 1 and programmed death ligand-1, and cytotoxic T lymphocyte-associated molecule-4 in hepatocellular carcinoma. Apmis.

[42] Wang JF, Wang YP, Xie J, Zhao ZZ, Gupta S, Guo Y (2021). Upregulated PD-L1 delays human neutrophil apoptosis and promotes lung injury in an experimental mouse model of sepsis. Blood.

[43] Johnson DB, Nebhan CA, Moslehi JJ, Balko JM (2022). Immune-checkpoint inhibitors: long-term implications of toxicity. Nat Rev Clin Oncol.

[44] Llovet JM, Castet F, Heikenwalder M, Maini MK, Mazzaferro V, Pinato DJ (2022). Immunotherapies for hepatocellular carcinoma. Nat Rev Clin Oncol.

[45] Erkekoglu P, Oral D, Chao MW, Kocer-Gumusel B (2017). Hepatocellular Carcinoma and Possible Chemical and Biological Causes: A Review. J Environ Pathol Toxicol Oncol.

[46] Chik F, Szyf M, Rabbani SA (2011). Role of epigenetics in cancer initiation and progression. Adv Exp Med Biol.

[47] Cheishvili D, Boureau L, Szyf M (2015). DNA demethylation and invasive cancer: implications for therapeutics. Br J Pharmacol.

[48] Toft M, Ross OA (2010). Copy number variation in Parkinson's disease. Genome Med.

[49] Ping Y, Zhou Y, Hu J, Pang L, Xu C, Xiao Y (2020). Dissecting the Functional Mechanisms of Somatic Copy-Number Alterations Based on Dysregulated ceRNA Networks across Cancers. Mol Ther Nucleic Acids.

[50] Yang XZ, Cheng TT, He QJ, Lei ZY, Chi J, Tang Z (2018). LINC01133 as ceRNA inhibits gastric cancer progression by sponging miR-106a-3p to regulate APC expression and the Wnt/β-catenin pathway. Mol Cancer.

[51] Liang H, Zhao Q, Zhu Z, Zhang C, Zhang H (2021). Long noncoding RNA LINC00958 suppresses apoptosis and radiosensitivity of colorectal cancer through targeting miR-422a. Cancer Cell Int.

[52] Mo Y, Lu Y, Wang P, Huang S, He L, Li D (2017). Long non-coding RNA XIST promotes cell growth by regulating miR-139-5p/PDK1/AKT axis in hepatocellular carcinoma. Tumour Biol.

[53] Wu J, Liu L, Jin H, Li Q, Wang S, Peng B (2019). LncSNHG3/miR-139-5p/BMI1 axis regulates proliferation, migration, and invasion in hepatocellular carcinoma. Onco Targets Ther.

